# Free functional muscle transplantation of an anomalous femoral adductor with a very large muscle belly: a case report

**DOI:** 10.1186/1749-7221-8-11

**Published:** 2013-10-28

**Authors:** Yukitoshi Kaizawa, Ryosuke Kakinoki, Souichi Ohta, Takashi Noguchi, Shuichi Matsuda

**Affiliations:** 1 Department of Orthopaedic Surgery, Graduate School of Medicine, Kyoto University, 54 Shogoin Kawahara-cho, Sakyo-ku, Kyoto 606-8507, Japan

**Keywords:** Free functional muscle transplantation, Brachial plexus injury, Adductor longus muscle, Gracilis muscle, Anomaly

## Abstract

We report the case of a 34-year-old man with a total brachial plexus injury that was treated by free functional muscle transplantation to restore simultaneously elbow flexion and finger extension. The muscle had a very large muscle belly (12 cm width), which was considered anatomically to be a fusion of the gracilis and the adductor longus muscles. Although the muscle possessed two major vascular pedicles with almost equal diameters, only the proximal vascular pedicle was anastomosed to the recipient vessels during the transplantation surgery, resulting in partial necrosis of the muscle. Several authors have reported on the successful simultaneous transplantation of the gracilis and adductor longus muscles, because they are supplied generally by a single common vascular pedicle. However, the present study suggests that when a surgeon encounters an aberrant femoral adductor with a very large muscle belly that can be considered to be a fusion of these muscles, the surgeon should assess intraoperatively the vascularity of the muscle using Doppler sonography, indocyanine green fluorescence injection, or other techniques.

## Text

### Background

The gracilis muscle has been used often for free functional muscle transplantation (FFMT) because of its long tendinous portion, its reliable vascularity with anatomical consistency, and the location of the nutrient vessels and innervating nerve in the terminal portion. We report the case of a patient with an aberrant muscle that was considered to be a fusion of the gracilis and adductor longus muscles. There are few reports of anomalies of the gracilis muscle [1]. To our knowledge, this is the first reported case of this type of anomaly, which we term an “adductor–gracilis muscle”.

### Case presentation

A 34-year-old man sustained multiple injuries including a total left brachial plexus injury (BPI) in a motorcycle accident. He was referred to us about five months after the injury for the treatment of a total palsy of his left brachial plexus and left accessory nerve. Six and a half months after the injury, his left brachial plexus was explored and its somatosensory evoked action potentials were studied. No response to stimulation of the cervical fifth (C5), C6, and C7 nerve roots was found, but there was a response to medial cord stimulation. Although Tinel’s sign was noted in the front axillary area at the time of exploration, functional recovery of the ulnar nerve was unlikely. We thus used the ulnar nerve vascularized by the superior collateral vessels as an interposition graft to connect the affected median nerve to the contralateral C7 nerve root (CC7 transfer [2]). For the restoration of elbow extension, the proximal ulnar nerve stump was connected to a branch of the radial nerve innervating the long head of the triceps brachii muscle. For the restoration of shoulder abduction, the median nerve with the medial cord contribution was connected to the axillary nerve.

The patient’s left elbow joint developed severe contracture because of heterotopic ossification, so three months after the nerve transfers, the patient received surgical resection of the ectopic bone and mobilization of his left elbow. The passive range of elbow motion was 120° in flexion and –20° in extension after a one-year rehabilitation exercise program.

**Figure 1:**
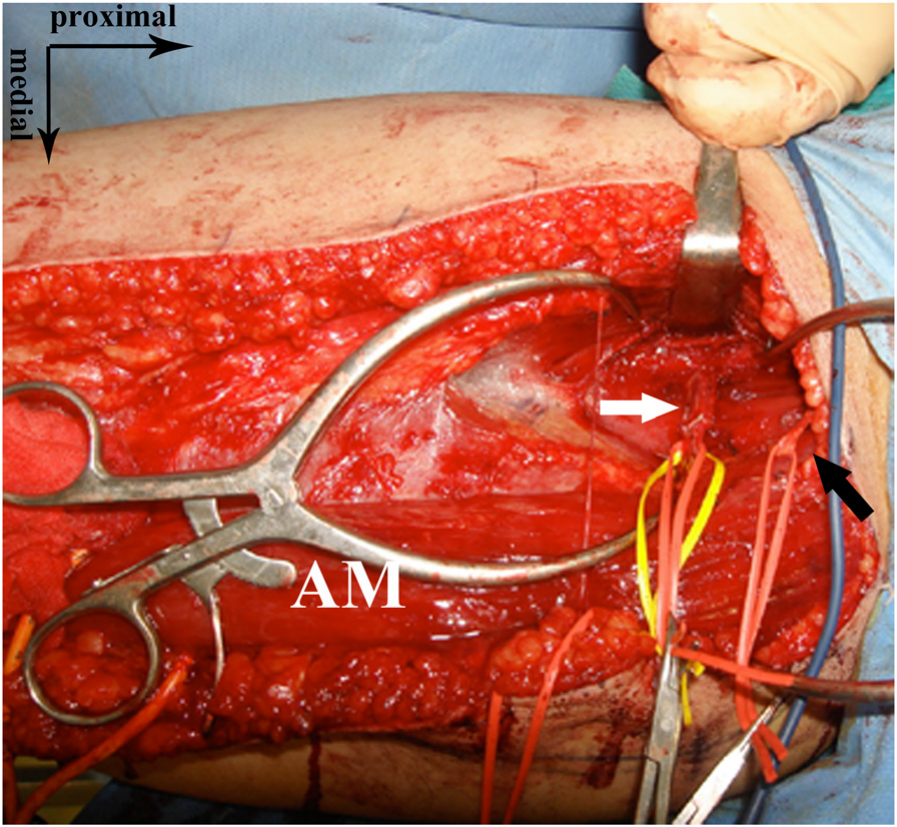
**Intraoperative view of the medial thigh.** The aberrant muscle with a very large muscle berry (AM) was found in the medial thigh. The adductor longus muscle was not identified. The deep femoral artery was exposed by retracting the gigantic muscle medially. White arrow indicates the proximal vascular pedicle and black indicates the obturator nerve.

**Figure 2:**
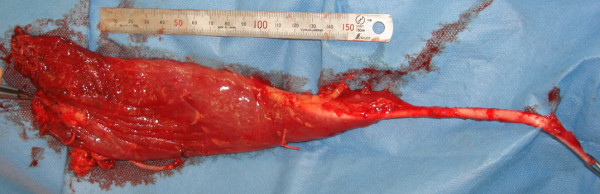
**Harvested aberrant muscle.** The harvested aberrant muscle considered to be a fusion of the gracilis and the adductor longus muscles was shown. The size of the muscle is smaller than that in situ because of loss of the muscular tension.

Two years after the nerve transfers, X-ray examination showed that his left shoulder was not subluxated. He had obtained left shoulder abduction and elbow extension to the M2 level of the Medical Research Council scale. At this point, we planned to restore his elbow flexion and prehension using a double free muscle transfer (Doi’s procedure [3]). For the first step of Doi’s technique, we identified a muscle with a very large muscle belly in the medial thigh, where the gracilis and adductor longus muscles should be located. The width of this large muscle was 12 cm, whereas that of the normal gracilis muscle is about 3.5 cm [4,5]. The deep femoral artery was exposed by retracting the large muscle medially (Figure 1). Based on this anatomical situation, we considered it probable that the very large muscle represented a fusion of the gracilis and adductor longus muscles (Figure 2).

There were two significant arteries nourishing the muscle. The proximal artery originating from the medial circumflex femoral artery entered the muscle at the center of the muscle belly, 6 cm caudal to the pubic tubercle. The distal artery, which was considered to arise from the superficial femoral artery, joined the muscle, 18 cm caudal to the tubercle. The diameters of both arteries were almost the same (about 2 mm). A branch of the obturator nerve, which looked anatomically normal, innervated the muscle. We wondered if the proximal vascular pedicle alone would be able to nourish the entire volume of this large muscle, but we were also concerned that anastomoses of both vascular pedicles at the recipient site would affect excursion of the muscle. Because several authors [6,7] have described the successful simultaneous transfer of the gracilis and adductor longus muscles with a common vascular pedicle, the distal vascular pedicle was ligated and the proximal pedicle was sutured to the left thoracoacromial vessels (one artery and one vein). Because of the persistent total paralysis of the patient’s left spinal accessory nerve, the muscle was innervated by a part of the vascularized ulnar nerve that had been transplanted in the previous CC7 transfer [8]. A monitor flap could not be attached to the muscle because using Doppler sonography we could not identify septocutaneous or intramuscular perforators from the nutrient vessels to the skin overlaying the muscle. The vascularity of the transplanted muscle was monitored by Doppler sonography and a visual check of the color of the muscle belly through a small skin window created over the muscle at the recipient site.

**Figure 3:**
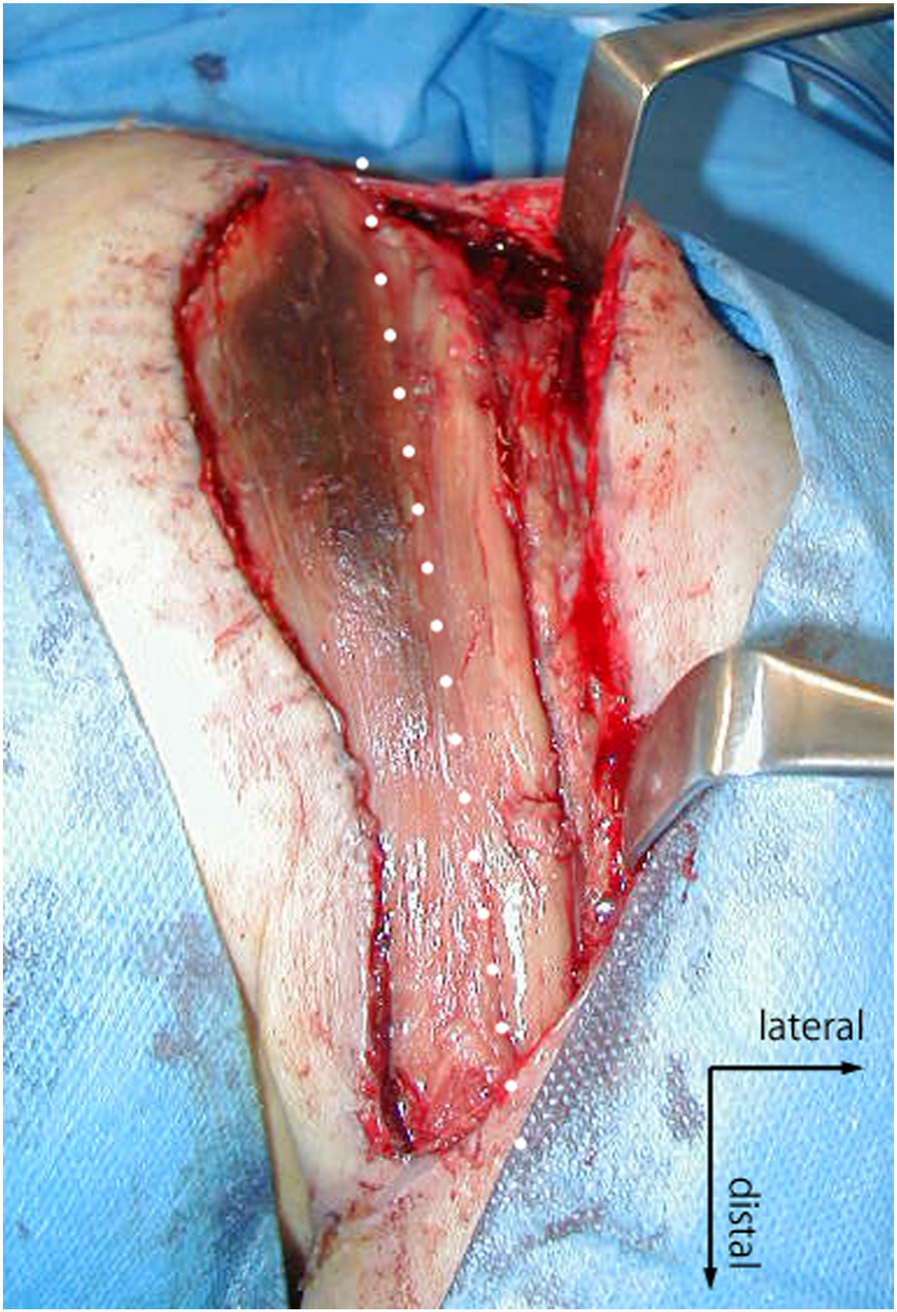
**Appearance of the transplanted muscle two weeks after the first FFMT.** Two weeks after the first FFMT, the wound was opened extensively in the operating theatre to irrigate the wound and check the entire muscle. The lateral one third of the transplanted muscle (right side of a white dot line) was found pale and fibrous, which meant the arterial insufficiency.

Two weeks after the surgery, a significant exudate was found in the wound. The wound was opened extensively in the operating theatre. Although the muscle color seen through the skin window was good and the sound of arterial blood flow was clearly audible with Doppler sonography, the lateral one-third of the muscle exhibited ischemic changes (Figure 3). The wound was infected by Staphylococcus epidermidis, which was treated with a course of antibiotics. We debrided the ischemic part of the transplanted muscle, from which no bleeding was observed. The skin defect in front of the shoulder region was covered by a pedicled muscle flap from the pectoralis major muscle (clavicular portion) and a lateral thoracic flap.

Eight months after the FFMT, because the sternal portion of the pectoralis major muscle had contracted significantly, the muscle was attached to the side of the surviving portion of the previously transplanted muscle to augment the strength of the muscle. Ten months after the first FFMT, a second FFMT [3] was performed for the restoration of finger flexion, using the left gracilis muscle, which was anatomically normal. The recipient vessels were the thoracodorsal artery and its corresponding vein. The recipient motor nerves were the fifth and sixth intercostal nerves. At the latest follow up, two years after the second FFMT, the patient has obtained M4 level motor function in his left elbow flexion and can use his hand with a hook grip.

### Discussion

Few anatomical anomalies of the gracilis muscle have been reported [1]. As far as we know, this is the first report of a muscle that can be considered to be a fusion of the adductor longus and gracilis muscles.

The gracilis muscle is classified as a Mathes–Nahai type II muscle [9] and is nourished by one dominant vascular pedicle and 1–4 minor pedicles [4,10]. There are intramuscular vascular anastomoses between the dominant pedicle and its adjacent minor pedicles [10]. The dominant pedicle of the gracilis muscle is usually located about 10 cm caudal to the pubic tubercle [4,5,10], and the entire gracilis muscle is nourished by this dominant pedicle alone [9,11]. The vascular and neural pedicles are long enough to allow muscle excursion, so the gracilis muscle is used frequently as a source for FFMT to treat facial nerve palsy [11], BPI [3], and Volkmann’s ischemic contracture [6].

Chuang et al. [6] reported a patient with Volkmann’s ischemic contracture who was treated with simultaneous transplantation of the gracilis and adductor longus muscles, with the anastomosis of a nutrient artery common to both muscles, to restore finger extension and flexion. Sananpanich et al. [7] studied the vascular anatomy of the gracilis and adductor longus muscles in cadaveric specimens, and found that in 98% of cadaveric specimens both muscles were nourished by a single common vascular pedicle. These studies indicate that simultaneous transplantation of the gracilis and adductor longus muscles can be performed successfully with a single anastomosis of the proximal vascular pedicle.

Our case suggested that anastomosis of only the proximal vascular pedicle might be insufficient to nourish the adductor–gracilis muscle, which looked like a fusion of the gracilis and adductor longus muscles. The muscle possessed two arteries, which were almost the same size, both of which may have contributed to nourishing the whole large muscle. The most likely explanation appears to be that the aberrant muscle in this patient might have had an intramuscular vascular anomaly, and that the lateral third might have been nourished only by the distal artery without intramuscular communication with the proximal artery.

When the surgeon encounters a very large muscle with this type of anomaly, we recommend that the surgeon assess the vascularity of the muscle before transplantation, using Doppler sonography, indocyanine green fluorescence video angiography [12], or other techniques. When an anomalous intramuscular vascularity is suspected, an alternative FFMT should be considered using the rectus femoris, latissimus dorsi, or other muscles.

### Conclusions

We report an aberrant femoral adductor with a very large muscle belly that was considered to be a fusion of the gracilis and adductor longus muscles. Simultaneous transplantation of the gracilis and adductor longus muscles nourished by a single common vascular pedicle is generally clinically feasible. However, our case suggests that, in this type of anomaly, the whole muscle is not vascularized completely by the proximal vascular pedicle, possibly because of the lack of an intramuscular vascular connection between the proximal and distal pedicles.

### Consent

Written informed consent was obtained from the patient for publication of this case report and any accompanying images. A copy of the written consent is available for review by the Editor-in-Chief of this journal.

## Competing interests

The authors declare that they have no competing interests.

## Authors’ contributions

YK performed all the pertinent literature reviews on the subject and wrote the manuscript. RK carried out the surgeries, reviewed the patient in the clinic, and collected the manuscript. SO and TN assisted RK in the surgeries. SM collected and helped draft the manuscript. All authors read and approved the final manuscript.

## References

[B1] Sainsbury JR Wagget J An absent gracilis–case report Br J Clin Pract 1984 8 72 6704300

[B2] Gu YD Zhang GM Chen DS Yan JG Cheng XM Chen L Seventh cervical nerve root transfer from the contralateral healthy side for treatment of brachial plexus root avulsion J Hand Surg (Br) 1992 8 518 521 1479244 10.1016/s0266-7681(05)80235-9

[B3] Doi K Sakai K Kuwata N Ihara K Kawai S Double free-muscle transfer to restore prehension following complete brachial plexus avulsion J Hand Surg 1995 8 408 414 10.1016/S0363-5023(05)80097-8 7642917

[B4] Taylor GI Cichowitz A Ang SG Seneviratne S Ashton M Comparative anatomical study of the gracilis and coracobrachialis muscles: implications for facial reanimation Plast Reconstr Surg 2003 8 20 30 10.1097/01.PRS.0000065909.86735.F7 12832872

[B5] Coquerel-Beghin D Milliez PY Auquit-Auckbur I Lemierre G Duparc F The gracilis musculocutaneous flap: vascular supply of the muscle and skin components Surg Radiol Anat 2006 8 588 595 10.1007/s00276-006-0150-8 17143568

[B6] Chuang DC Strauch RJ Wei FC Technical considerations in two-stage functioning free muscle transplantation reconstruction of both flexor and extensor functions of the forearm Microsurgery 1994 8 338 343 10.1002/micr.1920150510 7934802

[B7] Sananpanich K Tu YK Pookhang S Chalidapong P Anatomic variance in common vascular pedicle of the gracilis and adductor longus muscles: feasibility of double functioning free muscle transplantation with single pedicle anastomosis J Reconstr Microsurg 2008 8 231 238 10.1055/s-2008-1076096 18512203

[B8] Kakinoki R Ikeguchi R Nakayama K Nakamura T Functioning transferred free muscle innervated by part of the vascularized ulnar nerve connecting the contralateral cervical seventh root to themedian nerve: case report J Brachial Plex Peripher Nerve Inj 2007 8 18 10.1186/1749-7221-2-18 17883873 PMC2080628

[B9] Mathes SJ Nahai F Classification of the vascular anatomy of muscles: experimental and clinical correlation Plast Reconstr Surg 1981 8 177 187 7465666

[B10] Morris SF Yang D Gracilis muscle: arterial and neural basis for subdivision Ann Plast Surg 1999 8 630 633 10.1097/00000637-199906000-00008 10382799

[B11] Harii K Ohmori K Torii S Free gracilis muscle transplantation, with microneurovascular anastomoses for the treatment of facial paralysis. A preliminary report Plast Reconstr Surg 1976 8 133 143 10.1097/00006534-197602000-00001 1250883

[B12] Mothes H Donicke T Friedel R Simon M Markgraf E Bach O Indocyanin-green fluorescence video angiography used clinically to evaluate tissue perfusion in microsurgery J Trauma 2004 8 1018 1024 10.1097/01.TA.0000123041.47008.70 15580026

